# Recurrent Abdominal Pain Due to Acute Renal Failure With Loin Pain and Patchy Renal Ischemia After Anaerobic Exercise

**DOI:** 10.7759/cureus.63220

**Published:** 2024-06-26

**Authors:** Shiori Yazawa, Daisuke Matsuoka, Tsubasa Murase, Yoshiko Nakayama

**Affiliations:** 1 Department of Pediatrics, Minaminagano Medical Center, Shinonoi General Hospital, Shinonoi, JPN; 2 Department of Pediatrics, Shinshu University School of Medicine, Matsumoto, JPN; 3 Department of Pediatrics, Shinshu University Hospital, Matsumoto, JPN

**Keywords:** acute kidney injury (aki), hypouricemia, recurrent abdominal pain, anaerobic exercise, alpe

## Abstract

Acute renal failure with severe loin pain and patchy renal ischemia after anaerobic exercise (ALPE) is a rare condition characterized by severe loin pain and patchy renal ischemia following vigorous exercise. Moreover, its diagnosis relies on clinical manifestations. Here, we present the case of a 16-year-old male with recurrent abdominal pain attributed to ALPE. He developed recurrent abdominal pain after he started playing handball, and no definite cause could be identified despite a thorough examination. His symptoms worsened when he resumed handball practice after a one-month interruption. This case underscores the varied presentations of ALPE and the importance of considering it in the differential diagnosis of recurrent abdominal pain, particularly following strenuous exercise. Moreover, caution should be exercised when resuming exercise after periods of detraining, as this may predispose individuals to ALPE. Healthcare providers should be vigilant in recognizing and managing this condition, especially in individuals with recent exercise initiation following detraining.

## Introduction

Acute renal failure with severe loin pain and patchy renal ischemia after anaerobic exercise (ALPE) is termed acute kidney injury (AKI) induced by anaerobic exercise without myoglobinuria. The diagnostic criteria for ALPE are as follows [1]: (1) episodes of vigorous exercise (especially anaerobic exercise), (2) severe loin pain for 1-48 hours after exercise, and (3) serum creatine kinase (CK) level within the normal range or only slightly elevated (≤9 times the normal upper limit). Therefore, the incidental diagnosis of ALPE is unlikely, and clinical manifestations are crucial for its identification. We report a case of recurrent abdominal pain due to ALPE.

## Case presentation

A 16-year-old male with abdominal pain, nausea, and headache was admitted to our hospital. The patient’s medical history showed an irritable bowel syndrome diagnosis at seven years old. At 14 years old, the patient experienced recurrent abdominal pain and nausea approximately once a month. Since beginning high school at age 15, the patient started playing handball, increasing the abdominal pain frequency. The patient had undergone a thorough examination at our department two months before this episode, including endoscopy and ultrasonography; a clear cause was not identified despite a blood test result indicating hypouricemia (uric acid: 0.8 mg/dL) (Table 1). Due to an accompanying headache, abdominal migraine was suspected, and he was prescribed 300 mg oral acetaminophen as needed for abdominal pain. Subsequently, due to the COVID-19 pandemic, handball practice was suspended for one month. The patient had no family history of kidney dysfunction.

**Table 1:** Laboratory results ALPE, acute renal failure with severe loin pain and patchy renal ischemia after anaerobic exercise; N/A, not available

	Two months prior to the onset of ALPE	At the onset of ALPE	Reference range
Total protein (g/dL)	6.9	6.3	6.3-7.8
Albumin (g/dL)	4.7	4.3	4.1-5.1
Blood urea nitrogen (mg/dL)	15.7	38.6	6.8-18.8
Creatinine (mg/dL)	0.87	3.25	0.62-0.96
Uric acid (mg/dL)	0.8	5.2	3.7-7.0
Aspartate transaminase (U/L)	21	24	13-30
Alanine transaminase (U/L)	13	11	10-42
Gamma-glutamyl transferase (U/L)	14	16	9-48
Total bilirubin (mg/dL)	1.41	1.80	0.4-1.5
Creatine kinase (U/L)	61	427	50-275
Amylase (U/L)	61	48	44-132
Sodium (mmol/L)	143	140	138-144
Potassium (mmol/L)	4.7	3.3	3.7-4.7
Chloride (mmol/L)	109	105	102-109
Urinary occult blood	N/A	(-)	(-)
Urine protein	N/A	(-)	(-)
Urine ketone body	N/A	(-)	(-)
Urinary β2 microglobulin (μg/mgCr)	N/A	1.30	<0.30

Upon resuming handball practice, one day before hospital admission, he developed headache and nausea, later followed by severe abdominal pain and headache. He was prescribed acetaminophen, which was ineffective. The patient then visited our hospital.

His height and body weight were 166 cm and 50 kg, respectively. His vital signs were as follows: blood pressure, 119/73 mmHg; heart rate, 49 beats/minute; body temperature, 37.1℃; respiratory rate, 28 breaths/minute; and oxygen saturation (SpO_2_), 97% on room air.

Physical examination revealed severe tenderness in the left lower abdomen, which coincided with where the patient had been experiencing abdominal pain. There was no back pain, costovertebral angle tenderness, purpura, or abnormal skin turgor. Laboratory tests revealed a serum urea nitrogen level of 38.6 mg/dL, a serum creatinine level of 3.25 mg/dL, and an estimated glomerular filtration rate of 24 mL/minute/1.73 m^2^. The serum CK level was 427 U/L, and the uric acid level was 5.2 mg/dL. Urinalysis was negative for proteinuria, occult blood, and ketones and showed a urinary β2 microglobulin/creatinine ratio of 1.30 µg/mgCr (Table 1). The ultrasonography of the renal tract revealed the diffuse enlargement of both kidneys without stones or hydronephrosis.

Based on medical history, symptoms, and hypouricemia, ALPE was suspected. On the sixth day post admission, the abdominal pain resolved completely. Although the serum creatinine level worsened until the fifth day (6.39 mg/dL), it subsequently improved. When the serum creatinine level decreased to 1.62 mg/dL on the 10th day after admission, a delayed contrast-enhanced computed tomography was performed 24 hours after administering the contrast medium. The results revealed patchy, wedge-shaped, high-density areas in both kidneys, indicating delayed contrast excretion.

Based on these findings, a diagnosis of ALPE was confirmed. Although the patient was informed that vigorous exercise could cause ALPE, he strongly wished to continue playing handball. The patient was advised to avoid vigorous exercise if he felt any health-related discomfort, drink water when exercising to prevent dehydration, and avoid medications for the common cold or nonsteroidal anti-inflammatory drugs in general. He continued to play handball in high school without the recurrence of ALPE or abdominal pain.

## Discussion

This report describes the case of a patient with ALPE who experienced recurrent abdominal pain prior to ALPE diagnosis. In addition, the patient developed typical ALPE after an extended break from exercise, leading to the determination that ALPE was the cause of the recurring abdominal pain. Abdominal pain is frequently reported at the time of ALPE diagnosis [2,3]. Therefore, if symptoms caused by ALPE occur, creatinine levels would rise. This elevation would be evident on routine blood tests and would trigger the diagnosis of ALPE. However, diagnosing ALPE can be challenging when the patient is asymptomatic.

In cases involving children or adolescents, such as the present patient, wherein a series of examinations fail to identify organic abdominal disease, abdominal pain can lead to a differential diagnosis of functional gastrointestinal disorders such as functional dyspepsia, irritable bowel syndrome, abdominal migraine, and functional abdominal pain [4]. Therefore, when physicians suspect such diseases, they should also consider ALPE and confirm the relationship between abdominal pain and exercise in the interview.

Although the physiological mechanisms of ALPE remain unclear, failure to eliminate oxygen-free radicals is a hypothesis [5]. Plasma uric acid is a powerful antioxidant crucial for scavenging oxygen-free radicals generated during exercise and preventing renal ischemia resulting from renal vasoconstriction [6]. Hypouricemia incidence in patients with ALPE is more than 50 times higher than in patients without ALPE; 51% of reported ALPE cases have concomitant hypouricemia [7]. Furthermore, patients with ALPE with renal hypouricemia tend to develop severe hypouricemia (<1.0 mg/dL) after recovery from AKI, despite their serum uric acid levels being normal or only mildly increased during the AKI [2]. Therefore, assessing serum uric acid levels is useful for clinicians, especially for diagnosing ALPE in cases presenting with abdominal pain.

Our case also highlights the risk of developing ALPE when exercise is resumed after an extended period of detraining without an adaptation period. Long-term detraining decreases the capacity for aerobic metabolism and lowers the anaerobic threshold [8]. Specifically, the lactate threshold and muscle glycogen levels decrease, while respiratory exchange ratios increase [9]. Even after a five-week period of detraining, the levels of blood lactate, an end product of anaerobic metabolism, have been reported to increase fourfold from pre-detraining levels. It is well-established that oxidative stress escalates with increasing exercise intensity [10], indicating that oxidative stress intensifies post-detraining even at equivalent exercise intensities.

Moreover, the increased oxidative stress and decreased antioxidant capability after exercise have been linked to ALPE development [11]. Therefore, this oxidative imbalance could be further disrupted by decreased antioxidant efficiency due to long-term detraining, resulting in ALPE development despite the same exercise load. A similar case of ALPE after two months of detraining due to the COVID-19 pandemic has been reported [12]. Hence, returning to exercise after a few months of detraining carries the risk of developing ALPE.

## Conclusions

Clinical symptoms are very important in the diagnosis of ALPE; however, ALPE can present with a wide variety of clinical manifestations. Physicians should consider ALPE as a differential diagnosis for recurrent abdominal pain and use serum uric acid levels as a tool to help establish a definitive diagnosis. This is especially important for patients who have recently started exercising after a period of detraining, even if the exercise load remains unchanged.
